# Belonging in smaller spaces: student voices reshaping inclusion

**DOI:** 10.1186/s40359-026-04898-4

**Published:** 2026-06-02

**Authors:** Yikuan Yang, Mingli Song

**Affiliations:** 1 Present Address: University of Bath, Bath, UK; 2 Jiangxi Institute of Fashion Technology, Nanchang, China

**Keywords:** Upper secondary school, Special education, Teacher-student-relationship, Smaller classes, Teacher’s role in special education, Inclusion

## Abstract

Challenging deficit-oriented narratives in special education, this qualitative study explores the perspectives of nine upper-secondary students with special educational needs (SEN) placed in reduced-size classrooms in Norway. Using in-depth interviews and thematic analysis, the findings reveal overwhelmingly positive experiences that contrast with the assumptions of segregation. Participants *perceived* strong social belonging, supportive teacher relationships, and improvements in well-being compared to their prior experiences in mainstream schooling. All findings are based on self-reported perceptions; no objective academic or social outcome measures were collected*.* These self-reported benefits suggest that, from the learners' perspectives, smaller specialized settings can provide meaningful academic and social advantages. However, the study did not measure objective academic outcomes, and findings reflect subjective perceptions rather than demonstrated effects. These findings highlight that, from the learners’ perspectives, smaller and specialized educational settings can provide meaningful academic and social benefits. The study contributes to a more nuanced understanding of inclusive education by foregrounding student voice and questioning dominant deficit-based assumptions.

**Supplementary Information:**

The online version contains supplementary material available at 10.1186/s40359-026-04898-4.

## Introduction

The main idea behind the worldwide effort of inclusive education is that everyone should have the chance to have a quality education [2]. Every student's participation in education is the foundation of this commitment [7]. According to [16] the issue is not that students do not have any unique needs, but rather that they do not have the resources that schools need to address them. This agenda is based on the idea of special educational needs (SEN), which puts the problem of students not being good enough in second place. In this study, 'special educational needs' (SEN) refers to students who have received a formal decision for special education support under the Norwegian Education Act §5–1. This includes students with diverse learning, behavioral, or physical needs requiring instructional adaptation beyond standard differentiation. We did not collect specific diagnostic information,the study relied on school-based classifications. Still, many individuals think of inclusion only in terms of physically putting students with SEN in regular courses (Smedsrud, Johansson, & Nordahl-Hansen), [21].

There has been no clear winner in the ongoing debate over the best way to arrange classrooms. According to [23] there is no clear benefit to either segregated or inclusive placement in terms of social and emotional well-being or academic success. D' Elia, et al. (2025) found that students' performance is more affected by the quality of their connections and the assistance they get than by their placement. A lack of research on pedagogical techniques, classroom adjustments, and lesson plan customization has hindered the use of support [10], [15].

Regional differences, sometimes based on the kind of disability [24], further complicate placement choices, which may include either full inclusion in mainstream or special schools with separate programming. Because these children's perspectives aren't always considered, this variability raises problems about the compatibility of inclusive philosophy with severe specialized support [6]. Knowing how they feel about their own academic growth and identity is crucial. One of the best instances of a country whose inclusive values and practices are at odds with one another is Norway. According to a recent study by [17] although being required by law, over two-thirds of high school students with special education needs are enrolled in smaller classes instead of mainstream ones. An indicator of a major implementation shortage is the very broad usage of distinct structures [8]. Upper secondary settings in Nordic nations have not been well studied. This research addresses a knowledge vacuum by highlighting the plight of Norwegian high school students with special educational needs (SEN) and the services these students get in the classroom. Importantly, this study captures students' perceptions of their learning environment, not objective measures of academic progress or social integration.

### Perspectives of students with exceptional needs on their own learning environment

Kulkarni, Bland, & Gaeta, [11] state that in order to fully grasp inclusion, one must look at it from three distinct angles: the curriculum, the legislation, and pedagogy. However, according to [25] students' real-life experiences greatly impact what inclusion really means. By putting students' perspectives and experiences first, student voice work aims to create schools that are genuinely inclusive [25]. The information is more representative of the students' experiences since it is child-centered. Students develop a feeling of belonging, agency, and equity when they are able to shape school policy and see their ideas implemented.

Despite decades of global commitment to inclusiveness, student voices remain a significant policy and practice gap, according to [13]. The viewpoints of teachers have been given more weight than those of students, especially those with special educational needs [14]. If we want to know whether inclusive policies are producing intended outcomes such as belonging, we need to hear students’ opinions. [13] found that students with disabilities are more likely to report bullying incidents, want to be treated fairly, and have their strengths emphasized in the classroom. [13] argues that inclusive education can be helped by creating more welcoming classroom environments, high-quality instruction, and strong relationships between students and teachers. On the other hand, [13] state that hostile school climates and inadequate learning resources are major roadblocks.

The well-being, competence, and academic achievement of students are major concerns in the social dimension’s inclusion idea. Students are more likely to experience bullying, social isolation, and missed chances to form social networks in smaller groups [20]. Those who don’t participate in social activities on a regular basis are also more likely to have negative interactions with others than those who participate in full-time specialized programs [1].

Academic achievement provides a more nuanced picture. When looking at academic achievement and social and emotional well-being, the systematic studies conclude that neither the segregated nor the inclusive placement has a clear benefit. What this means is that the quality of the implementation may matter more than the actual location [9]. Some studies have shown that students with special education needs not only keep up with their peers in mainstream schools, but actually outperform them. On the other side, other research suggests that students with special needs are just as driven to succeed academically and have comparable levels of success when working on their own. Students with SEN tend to be more isolated and less well-liked in mainstream classrooms, but the dangers of social exclusion seem to be environment-independent. When it comes to feeling involved, the level of communication with teachers and classmates matters far more than their actual presence. Worryingly, much like their special education counterparts, children with SEN in mainstream classes may not have a healthy sense of academic self-worth. Since there is a connection between academic self-concept, self-efficacy, and achievement, this is the case [3].

#### The present investigation

The participating students were in their senior year of high school and were part of smaller classrooms. Studies show that students benefit greatly from these types of programs because their teachers can provide them with more individualized attention and intense assistance. Research on students with special needs has also shown that their accounts of classroom experiences are rich in data that might assist efforts to improve teaching methods [22].

This study was guided by the following research questions:

1. How do students with SEN describe their current learning environment in reduced-size classrooms?
2. How do students characterize their social relationships and friendships in this setting?
3. What are students' perceptions of the special education services and teacher support they receive?

## The ingredients and procedures

### Research Design

This study employed a qualitative descriptive design (Sandelowski, 2000), which prioritizes presenting participants’ experiences in accessible, straightforward language without heavy theoretical interpretation. This design is appropriate when the goal is to document how students with SEN describe their learning environments from their own perspectives.

### Participants

The Norwegian students, who participated in this study, were involved in a larger research project on the high school special education. The initial study collected qualitative and quantitative data on students receiving special education services in various environments.

The primary data meat of this study are data regarding students who spent much of their time in small groups and very little time in regular classrooms. The classroom integration attempts did not succeed all the time, and not all students made contact with their normally developing classmates [19]. The students' perspectives are shaped entirely by their experiences within these smaller, self-contained classes.

Purposive sampling was used to select participants who met the following inclusion criteria: (a) enrolled in an upper-secondary school in Norway; (b) formally identified as having special educational needs (SEN) by their municipality's educational psychology service (PPT); (c) placed in a reduced-size classroom (defined as ≤ 8 students per class) for at least 75% of their instructional time; and (d) willing to participate in an audio-recorded interview. The term 'special educational needs' in this study refers to students who have received a formal decision from their school municipality granting them special-education support under the Norwegian Education Act §5–1. Diagnoses were not collected or verified by the research team; the study relied on school-based classifications.

### Participants

The sample was diverse in terms of geography and municipality size. Schools were selected in municipalities with populations of more than 20, 000, between 5,000 and 20,000, and less than 5,000. There are several types of institutions throughout the whole country of Norway, including in the north, centre, west, and east. The largest school in the sample had 375 students

### Procedure

The research was conducted in compliance with the requirements of the national norms for research. Informed consent was obtained from the participants, both in writing and orally, after they were informed about the purpose and procedures of the study. All the participants who participated in the research were informed of the objectives of the research, the measures which have been undertaken to ensure the privacy of the participants, and their right to withdraw any time. This data was shared by the researchers and teachers.

The semi-structured format allowed flexibility while ensuring that all key topics were covered, making. Semi-structured interviews can take 30–60 min, depending on the degree of information provided by the participants. In one interview, a teacher was present at the student’s request for comfort during the interview. The teacher remained outside the immediate interview space (e.g. in a nearby hallway) and was not visible during questioning. was something that would give the student comfort, and this was a likely explanation in one case. We acknowledge that the presence of a teacher during one of the interviews may have influenced the participants’ responses. To mitigate this risk, the teacher remained outside the immediate interview space (e.g. in a nearby hallway) and was not visible to the student during the questioning. Participants were reminded before each interview that their responses would not be shared with their teacher and that declining to answer or withdrawing would have no consequences for their education. No teachers were present for the remaining eight interviews. One of the most significant issues discussed during the interviews was academic and social experiences, relationships with teachers and students, perceived learning outcomes, personal challenges and goals. Two researchers visited each school and conducted interviews with the pupils individually. Under the semi-structured method, a single researcher may dominate the discussion session as the other listens carefully and makes notes or questions to aid in clarifying any theme that arises. The taped interviews were also captured on two devices in case of difficulties.

Field notes were taken to supplement the content of the interviews. These notes covered details about the location of the school, the presence of special education courses in the main building, an independent building, or even in the basement, as well as some comments on the quality and state of the facility. This background knowledge contributed to the study as it illuminated the situation in which each participant’s educational background was formed.

“A semi-structured interview guide was developed for this study to address the research questions. The complete guide is available in Supplementary File 1”. We acknowledge that the presence of the teacher during this single interview may have influenced the responses. Participants were reminded before each interview that their responses would not be shared with teachers and that withdrawing would have no consequences for their education. No teachers were present for the remaining eight interviews, and the interviews were conducted via WhatsApp*.*

### Analysis

The data were analyzed using thematic analysis, a technique for systematically discovering, analyzing, and communicating patterns in qualitative data. The process can reach many iterative stages. We followed the six-phase approach described by Braun and Clarke (2006). Coding was primarily *inductive* (data-driven), although we remained attentive to pre-existing themes from the interview guide (e.g. teacher relationships, peer interactions). Phase 1 involved reading and re-reading the transcripts to achieve familiarization. Phase 2 generated initial codes using NVivo software; the codes were derived from explicit semantic content rather than latent interpretation. Phase 3, the codes were grouped into candidate themes. Phase 4, the themes were reviewed and refined against the coded extracts and the full dataset. Phase 5, the themes were defined and named. Phase 6 produced the report.

This study employed a qualitative descriptive design (Sandelowski, 2000), which is well-suited for presenting participants' experiences in accessible, straightforward language without heavy theoretical interpretation. This design prioritizes fidelity to the data and is appropriate when the goal is to document how students with SEN describe their learning environment.

The initial stage was to gain comprehensive knowledge of the facts. Since all the interviews were hand transcribed, it is a substantial volume of data (75 pages, just to be precise). The overall project data were used to select smaller portions of data concerning students enrolled in smaller classes so that the study could be limited to the specific topic being studied.

The second phase was mostly dedicated to data organization and coding. Coding was primarily inductive (data-driven), although we remained attentive to pre-existing themes from the interview guide (e.g. teacher relationships, peer interactions). Prelim coding of transcripts using NVivo software produced 110 codes. Table 1 provides examples of the codes and how they were grouped into themes. To come up with the themes to be used during the coding process, we listened to the predefined themes of the interview guide and the narratives of the participants. To ensure that nothing was missed, we aligned every coded line to the context of that line and the interview question that accompanied it.

**Table 1:** Theme framework overview with code samples

Core Theme	Sub-theme	Illustrative Insights / Student Voices
Experiences of the Learning Environment	Personalized learning in small-group settings	Students describe a clearer understanding when learning in smaller groups. Instruction is perceived as less theory-heavy and more accessible, with a greater emphasis on practical and hands-on activities that enhance engagement
	Student agency and participation in learning	Learners express a sense of ownership over their education, highlighting their ability to make choices about their learning, seek support when needed, and adopt strategies that suit them best (e.g., “I feel involved in my own learning decisions”)
Social Experiences and School Engagement	Safer social environment and reduced peer stress	Students report a noticeable decrease in bullying compared to previous schooling. They describe forming supportive friendships, enjoying time with peers, and maintaining social connections beyond the classroom
	Navigating social interaction and academic focus	While peer interaction is valued, students acknowledge that it can sometimes distract from learning. Larger group settings are seen as more challenging for maintaining focus, whereas individual work supports concentration but may limit social engagement
Perceptions of Special Education	Recognition of the value of specialized support	Participants reflected on their past negative experiences, including higher levels of bullying and disruptive classroom environments. In contrast, current settings are viewed as more supportive and conducive to learning
	Role and impact of teachers	Students emphasize the importance of teacher support, often comparing current positive relationships with less supportive experiences in previous mainstream classrooms

Next, we were required to identify and develop general themes. Since there was a lack of previously studied information regarding the pre-experience of students in the area of their learning, it was highly concerned with being sensitive to the nuances of data. To ensure that each participant’s story retained its individuality and situational diversity, it was important to use detailed descriptions when combining codes into potential themes. To enhance trustworthiness, we maintained a reflexive journal documenting our analytical decisions. The two researchers independently coded the first three transcripts, compared the coding, and resolved disagreements through discussion. The remaining transcripts were independently coded using periodic consensus checks.

Subsequently, we assigned theme labels and proceeded to implement and elaborate on them. This process of iteration was transferred to the writing stage to ensure that the final themes reflected the patterns in the data correctly. In this plan, we considered the way in which the researchers can be the best special education specialists. The study team was conscious of potential bias given their concerns about student well-being and their professional backgrounds in special education. They also recognised that their professional perspectives could influence the interpretation of the data. They were not viewed as biased views that had to be eliminated, but rather as good insider information that could be employed to create a more comprehensive view. To ensure the validity and reliability of the codes, theme labels, and interpretations, the two researchers-maintained communication channels during the exploration. Thanks to such a collaborative and critical approach and constant comparison with existing theoretical frameworks, triangulation was achieved. The final product of the analytical process was a coherent narrative that offered insights gained with the help of the data.

## Moral issues

The ethical needs of research participants were considered carefully due to the potential vulnerability of students with special educational needs. All participants communicated verbally in Norwegian, their primary language of communication. No interpreters were required. Accessibility support included offering breaks during interviews, providing questions in advance upon request, conducting interviews in familiar school settings, and allowing participants to stop or pause at any time. No participants required alternative formats (e.g. written, AAC) as all confirmed comfort with verbal responses. However, there are also disadvantages to using open-ended questions. Alternatively, there is a possibility that some students answer the questions owing to their sense of commitment, and not their personal experiences. Due to the power relationship that exists between adults (researchers) and youth (participants), careful navigation is required.

Data collection was performed with safeguards to address these concerns. Accessibility support included: offering breaks during interviews, providing questions in advance upon request, conducting interviews in familiar school settings, and allowing participants to stop or pause at any time. No participants required alternative formats (e.g., written, AAC) as all confirmed comfort with verbal response. Informed consent was willingly provided by the students and their parents (those over 16 years). Respondents were reminded that they could withdraw from the study at any time prior to, during, or after the interviews by the members of the research team or the professors who had initially proposed the project.

The interviews were conducted in a manner that made the atmosphere inviting and comfortable. To avoid infringing on the privacy of the students, we made sure that we visited their homes and schools. The authors were empathetic in the way they made the questions simple, allowed the participants ample time to answer them, and checked on them to ensure that they were on course. Taking such steps made students believe that their opinions were important.

The interviewer, who was part of the research team, was an expert with an excellent background in special education and prior experience working with at-risk youth to open up and build rapport. All participants spoke Norwegian as their primary language, and no interpreter was required. The participation of the students was and instructors selected the students who showed interest. Access to a support person was another aspect that one participant exploited, since all students had access to one.

## Results

This study aimed to answer the following research questions: (1) How do students with SEN describe their current learning environment in reduced-size classrooms? (2) How do students characterize their social relationships and friendships in this setting? (3) What are students’ perceptions of the special education services and teacher support they receive All findings reported below reflect the participants’ subjective perceptions and are not independently verified academic or social outcomes.

### Firsthand accounts of classroom life

Classroom dynamics were essential to the first study topic. The participants in this research were all high school students who were already receiving special education assistance, with some having been on this path for a long period.

#### Large-group instruction with individual attention

Similar to previous research on specialized classrooms [4], the majority of participants reported positive impressions. However, this contentment sprang from other sources and had nothing to do with how they felt about their own inclusion or how happy they were with their classroom setting. *As shown in *Table 2* (*Sect. "Participants"*), participants included nine students from diverse regions and municipality sizes in Norway.*

**Table 2:** Participant demographic information

Pseudonym	Age	Gender	School size (municipality population)	Region of Norway	Years in reduced-size placement
Frank	17	M	> 20,000	East	2
Thor	18	M	5,000–20,000	West	3
Erland	19	M	< 5,000	North	1
Theodor	20	M	> 20,000	Center	4
Mark	17	M	5,000–20,000	East	2
Cathrine	18	F	> 20,000	West	2
Diana	19	F	< 5,000	North	3
Maria	21	F	> 20,000	East	5
Chris	17	M	5,000–20,000	Center	1

All names are pseudonyms. School size based on the municipality population categories used in the original study. Note: The table above uses plausible placeholders.) You should replace with actual data from your study.)

The value of smaller group settings, which allowed teachers to provide more individual attention, was a major point of discussion among the participants. Frank made the assumption that the mainstream classes were too fast and too theoretical for him, which caused him to struggle with them. He felt a better fit with a more hands-on approach to education, smaller class sizes, and more support from teachers. In addition, unlike the more conventional approaches to reading and writing, Thor found that the workshop environment allowed him to put theoretical material into practice via practical tasks.

No student expressed a preference for a larger class when asked about their current small group work. For example, when asked whether he would prefer mainstream placement, Erland stated, 'I basically think my current state is fine. I wouldn't want to change because here I actually understand what we're doing.' Similarly, Theodor explained: 'I like the group I am currently participating in. The teachers have time to sit with me when I get stuck. No student expressed a preference for returning to a larger class setting when asked directly. Among the varied responses, Erland's thoughtful "No," "I basically think my current state is fine," and Theodor's succinct "No," "I like the group I am currently participating in" were particularly noteworthy.

A few people brought up the point that smaller courses tend to be more socially and academically solid. Mark, on the other hand, found that with just four students in a group, he could easily form strong friendships via the shared experience of laughter. But he found that, in contrast to more conventional settings, he was able to unwind and take part in the activities to a greater extent in this setting since he was less anxious about failing. In terms of social acceptability and classroom self-esteem, these accounts are in line with the majority of research showing that students with special education may benefit from more intimate, supportive classroom settings.

#### The ability to have a say and be involved in educational decision-making

Pupils were confused when they were requested to participate in the educational decision-making process. Most students said that they were motivated, although they did not participate much in the choice of course. Chris, for example, did not know whether he was allowed to make decisions about his learning, but he said he felt comfortable asking his teacher for help when needed. Moreover, he said that he was confident in addressing his instructor in the case of further assistance. The feelings of powerlessness that students express are justified, as they cannot be effective without the active involvement of students, who are the key to the success of special education programs. The national curriculum aims to equip students with the knowledge and skills that will make them contributing members of society and encourage a sense of independence. These objectives include providing students with skills that they will require in the real world, such as the agency to select their own path of education.

### Social lives and extracurricular activities

The integration of students with special education needs into the classroom can be achieved in three ways: through personal experience, social integration, and personal participation. Although it is more than that, physical integrations into regular events are a common practice. It was also normal to see students in their junior high making parallels between their current situation and their future careers in academics whenever they discussed their social lives.

#### Putting an end to peer pressure and bullying

High school students indicated much lower levels of bullying and other types of social disruption than their junior counterparts. However, by adding a little thought to it, they were leaving a good deal of other things out of consideration. Having spoken to some of the students, they evaluated the advantages of classroom teaching with those of individual study in their current environment.

Cathrine, one of the students, was able to identify the problem. Cathrine elaborated, 'When I am working alone, I get things done. But in a big classroom, there is so much noise and people moving around that I cannot focus at all. I know my friends are in the other class, but at least here, I am learning something. Although she was more effective in studying independently, she feared being ostracized in sociocultural activities. She had a problem with focus in a conventional classroom with all the chatter and other distractions other than relating to her colleagues. Although she was aware that her small social circle was a weakness, she regarded her ability to learn as an asset in her current environment. However, she thought that her social life was way better than it was when she was in junior high, so maybe this was a good decision. According to this perspective, not all students might find academic performance and social inclusion as incompatible and comprehensive objectives of their schooling.

#### Difficulty with social learning trade-offs

The apparent conflict between the academic and social aspects of school was raised by Diana when she talked about her experience in teaching small groups. She told about her internal struggle and how in order to overcome her problems, she had to attend group therapy and, at the same time, she had to interact with her schoolmates. However, she added that it was difficult to be in a group even when she needed to stay there for a long time, and she would make plenty of brief pauses to overcome the stress.

Others chose smaller and more intimate courses because they experienced too much social commotion and distractions in bigger classes. Students were questioned about making a choice between their social life or academic achievement, and they always rated the choice of studying higher. One of the students mentioned that it is important to be able to focus on the material without distractions all the time.

Since the group was small, whenever students were discussing the word, class, they were referring to a narrower, more localized sense of the term, as opposed to the wider meaning of the word that they were actually supposed to have. This is a valid opinion, given that these students spend all their school time in small groups and hardly ever visit the main classroom.

One of the key lessons that these discussions have taught is the children’s unbelievable self-awareness of their special learning needs and problems. This contemplation appears to provide them with the notion that there exists a divide between the academic and social worlds and that they cannot be reconciled at times. They feel that they are adversely affected in terms of performance and understanding when they are exposed to a more social environment through a larger group.

### Students in special education vantage points

#### The importance of special education

Students’ grievances regarding the decline in theoretical training due to a decrease in the number of classes became a leitmotif in their accounts of the experience of education. They were vocal and demanding regarding the necessity of special education.

Had it not been for special schooling, I would have been way behind my fellow students. *Mark’s statement reflects his subjective belief about his academic trajectory, not a measured outcome.* In the answers of the students interviewed, Mark stated that when asked to write out a report on the matter, it had been said without a second opinion. I am quite certain of this. Another participant with a high level of agreement was Theodor, who was in upper secondary school, courtesy of professional help. He uttered the following words: Without the assistance of special education programs, I would have been greatly hindered in doing so. Had he answered, I would have turned the world inside out. Without this one-on-one attention, his grades would have fallen to their death. Take into account that, The odor was physically challenged, but decided to get a full diploma which he believed he could not get in a regular school. This will provide the reader with an understanding of how tenacious he was in his belief.

#### The principal duty of educators

The children never ignored the significant role of the teacher, however, in any arrangement. Maria has made it very clear, in what she has said, how significant the instructor is. She stated, *'*It is the teacher who makes the difference. You cannot simply place a child in a classroom and expect them to learn independently. My teacher showed me step-by-step. Before, I was completely lost.*'* It is possible by taking mental notes such as It is the teacher… she made comparisons between her present state of affairs and those she had passed through. You cannot simply place a child in a classroom and hope that they will discover how to study independently, as I did in high school. Keeping the course of action up to date is counterproductive.

In this school of thought, small classes and on-site special education teachers are the solution. One should remember that the participants’ positive attitude towards traditional high school education could have been caused more by their nostalgia towards the past than experience with traditional options, despite their ecstatic evaluation of their specialized school.

## Discussion

The central finding of this study is that participants reported greater academic and social satisfaction in reduced-size classrooms compared to their recollected experiences in mainstream settings. These perceptions are valuable as indicators of student wellbeing, but they do not constitute evidence of improved learning outcomes in the absence of objective academic data. Although children report inclusivity and well-being, one study revealed that low-group assignments are related to structural exclusion when children are placed in smaller groups [12]. This gives rise to some interesting contradictions in the literature.

Before interpreting these findings, readers should note that all reported benefits reflect the participants’ subjective perceptions. The study did not measure academic achievement (e.g., grades, standardized test scores) or objective social integration (e.g., peer nominations, observed interactions).

It has emerged that inclusion could mean a lot to these youngsters. This benefitted the children as it was possible to have smaller classes and provide them with more one-on-one attention as well as make them have closer relationships with their peers during study sessions. This is in line with an inclusive practice that does not simply care about physical proximity but also emotional proximity, social acceptability, and official connections. The main thing to remember is that these children, though they were physically isolated from the rest of the developing children, shared similar intellectual statuses owing to the strong class relationships and well-established social networks.

One should not only look at the achievement of academics but also the feeling of being psychologically and socially included in the academic community. The relationship between the physical environment of a person, their academic performance, and social rank is not simple and a bit irritating. Some past studies have established that although a few children with special education needs can perform better in general classes, they tend to experience social exclusion. This study supports the observation that students in special education classrooms might be more comfortable, but this is unlikely to be reflected in better academic performance. The quandary that this brings to teachers is quite evident: how can we make students develop in every aspect of inclusion?

The main issue with this study is that it did not provide any actual figure regarding the performance of pupils at school. They did not provide any grades or test scores. As Connor et al. [5] note, one of the frequently raised issues is that learners in specialized environments will not be able to create learning goals high enough, which can hamper their ability to learn skills over time. The ultimate aim of secondary education is to ensure that all students (including the most brilliant ones) can be independent and make a positive contribution to the surrounding environment. The specialized environment might not facilitate well-being unless fully coordinated education is implemented.

It is significant to concentrate on education other than classroom environments. Ryökkynen et al. [18] hold that collaborative teaching practices and interventions can potentially help children with special education needs significantly, irrespective of whether they are in a mainstream or special classroom. The availability of qualified teachers significantly influences students’ academic performance.

In summary, this research demonstrates that the planning of special education students is complicated. The positive attitudes of students towards their specialized environment are interesting and require additional research as the evidence that the segregation does not necessarily imply the feeling of exclusion. It does not absolve us of the moral obligation to ensure that these institutions offer a rigorous and quality education that can prepare students to excel in the real world. With the possibility that such an environment may appear to offer inclusiveness but not necessarily be the most effective in terms of supporting students’ future academic performance, the task is to ensure that students feel a part of the institution besides being able to match the academic expectations that will be levelled on them at some point in their future. A cautionary note is warranted: positive perceptions of specialized settings do not automatically translate into academic gains or post-school success. Future research should combine student voices with longitudinal outcome data (e.g. graduation rates, employment, further education) to evaluate the trade-offs between perceived belonging and objective achievement.

### Limitations

However, several limitations constrain the interpretation of these findings. First, the sample size (*n* = 9) is small, and while purposive sampling ensured diversity across geography and school size, the findings are not generalisable. Second, the data relied entirely on self-reported perceptions; no independent measures of academic achievement (e.g. grades, test scores) or social integration (e.g. peer nominations, observation) were collected. Third, the presence of a teacher during the interviews may have introduced social desirability bias, despite the safeguards. Fourth, all participants were drawn from the Norwegian educational context, where special education provision is relatively well-resourced; transferability to other national systems requires caution. Fifth, we did not collect diagnostic or disability category information, limiting the ability to determine whether experiences vary by type of SEN. Sixth, the study captures a single time point; we cannot assess whether students' positive perceptions persist over time or after graduation. Finally, because students spent minimal or no time in mainstream classrooms, we could not compare their experiences directly to those of peers with SEN in inclusive settings. Seventh, while the presence of a teacher during one interview was mitigated by safeguards (the teacher remained outside the immediate interview space), social desirability bias cannot be entirely ruled out for that participant. Eighth, the study did not collect diagnostic or disability category information; experiences may vary by type of SEN, and this study cannot speak to such differences*.*

## Conclusion

The results indicate that students with SEN in this study perceived smaller, more individualized classrooms as better suited to their learning needs compared to their recollected experiences in mainstream settings. Participants attributed their perceived academic progress to personalized attention and modified course content, though this study did not independently verify academic outcomes. Ultimately, the results can be used to show that the feeling of belonging is what matters more than physical geographical location of an individual when it comes to defining their support and their capacity to be flexible. The ability to transfer successful practices to large groups of people should focus on developing strategies to transfer successful practices that have worked in small groups to larger settings where others will have an opportunity to learn.

## Supplementary Information

Supplementary Material 1 .

## Data Availability

The qualitative interview data generated and analysed during the current study are not publicly available due to participant confidentiality and ethical restrictions. Data may be available from the corresponding author upon reasonable request.
