# Supplementary material for: Belonging in smaller spaces: student voices reshaping inclusion
Source: BMC Psychol. 2026 Jun 2;14:1138. doi: 10.1186/s40359-026-04898-4 (PMC13445814; doi:10.1186/s40359-026-04898-4)
Supplement: Supplementary file 1 [file 40359_2026_4898_MOESM1_ESM.docx]

**Supplementary File 1: Interview Guide**

**Title: Semi-Structured Interview Guide**

**Introduction**

Thank you for participating in this study. We are interested in understanding your experiences in your current learning environment, especially in smaller classes. There are no right or wrong answers. Please feel free to share your honest thoughts. You may skip any question or stop at any time.

**Section A: Background and General Experience**

1. Can you tell me a little about your current school and class?
2. How long have you been studying in this type of classroom (small group/special class)?
3. How would you describe your overall experience in your current learning environment?

**Section B: Learning Environment and Academic Experience**

4. How do you feel about learning in a smaller class compared to larger classes?
5. What differences have you noticed between your current class and previous mainstream classes?
6. Do you find it easier or harder to understand lessons in this setting? Why?
7. Can you describe the teaching methods used in your class (e.g., practical work, theory, individual support)?
8. How do teachers support your learning in this classroom?
9. Do you feel you are able to participate in your own learning decisions? Can you give an example?

**Section C: Social Experiences and Relationships**

10. How would you describe your relationships with other students in your class?
11. Have you made friends in this learning environment? Can you describe these friendships?
12. How does this classroom affect your social life compared to your previous school experience?
13. Have you experienced bullying or negative interactions in the past or present? If yes, how does it compare?
14. Do you feel comfortable and safe in your current class? Why or why not?

**Section D: Perceptions of Special Education**

15. What do you think about the support you receive in this class?
16. How has this type of education helped you academically?
17. Do you think you would have performed differently in a regular (mainstream) classroom? How?
18. What are the advantages of being in a smaller/special class?
19. Are there any disadvantages or challenges?

**Section E: Teacher Role and Support**

20. How would you describe your relationship with your teachers?
21. In what ways do teachers help you feel supported or included?
22. Can you compare your current teachers with those in your previous schools?

**Section F: Personal Development and Future Outlook**

23. How has this learning environment affected your confidence or motivation?
24. Do you feel more included or that you belong in this setting? Why?
25. How do you think this experience will affect your future (education, career, life)?

**Closing Question**

26. Is there anything else you would like to share about your experience that we haven’t asked?
